# Multi-modal connectome analysis of the stereoelectroencephalography implantation effect: a case report

**DOI:** 10.1016/j.cnp.2026.09.002

**Published:** 2026-09-12

**Authors:** Taichi Sayanagi, Kenzo Kosugi, Takuya Enomoto, Masashi Fujisawa, Shiho Matsuoka, Junko Shinohara, Toshiki Tezuka, Yuji Nakayama, Masahiro Toda

**Affiliations:** a Department of Neurosurgery, Keio University School of Medicine, 35 Shinanomachi, Shinjuku, Tokyo, Japan; b Department of Clinical Laboratory, Keio University Hospital, 35 Shinanomachi, Shinjuku, Tokyo, Japan; c Department of Neurology, Keio University School of Medicine, 35 Shinanomachi, Shinjuku, Tokyo, Japan; d Department of Pathology, Keio University School of Medicine, 35 Shinanomachi, Shinjuku, Tokyo, Japan

**Keywords:** Epilepsy, Stereoelectroencephalography, SEEG, Connectome, Diffusion MRI, Functional MRI

## Abstract

Stereoelectroencephalography (SEEG) may induce seizure reduction in some patients, known as the implantation effect, but its network mechanism remains unclear. We investigated whether SEEG electrode insertion is associated with measurable brain network reorganisation using longitudinal multi-modal analysis. We studied a patient with drug-resistant right temporal lobe epilepsy who experienced seizure reduction after SEEG implantation and subsequently underwent anterior temporal lobectomy, achieving ILAE class 1a seizure freedom at 6-month follow-up. Structural connectivity from diffusion MRI, resting-state functional connectivity and intracranial EEG connectivity were analysed longitudinally. Connectome gradients were computed using diffusion map embedding, and resected tissue was examined histopathologically. Multi-modal analysis showed convergent connectivity changes after SEEG implantation: decreased hippocampus–amygdala connectivity, decreased fusiform gyrus–temporal pole connectivity and increased hippocampus–posterior cingulate connectivity. Diffusion MRI gradient analysis showed eccentricity reduction of 11.9% after SEEG and 17.6% after lobectomy, consistent with greater network integration. SEEG connectivity showed reduced limbic synchronisation between days 3 and 7 of monitoring. Histopathology demonstrated lymphocytic infiltration and reactive gliosis around electrode trajectories, consistent with chronic inflammation. SEEG implantation was associated with limbic disconnection, enhanced hippocampus–posterior cingulate connectivity and partial normalisation of connectome topology. The convergent findings suggest that electrode-induced micro-lesions may produce network-modifying effects that contribute to the implantation effect.

## Highlights

• Limbic network connectivity changed after stereoelectroencephalography (SEEG) alone.
• Functional and structural MRI also showed limbic disconnection after SEEG.
• SEEG alone resulted in two-thirds of the gradient shift later achieved by lobectomy.

## 1 Introduction

Stereoelectroencephalography (SEEG) is essential for defining the epileptogenic zone in drug-resistant focal epilepsy, particularly in complex or MRI-negative temporal lobe epilepsy (Jayakar et al., 2016; Suresh et al., 2015). Seizure reduction after intracranial electrode implantation alone—the implantation effect—has been reported after SEEG and subdural monitoring, and is generally attributed to a micro-lesion effect caused by electrode insertion (Bottan et al., 2023; Katariwala et al., 2001; Kaur et al., 2019; Percy et al., 2020). However, whether this clinical phenomenon is accompanied by measurable brain network reorganisation remains unknown.

Here, we report a patient with drug-resistant temporal lobe epilepsy who experienced seizure reduction after SEEG implantation and achieved seizure freedom after subsequent anterior temporal lobectomy. Using longitudinal diffusion MRI, resting-state fMRI, intracranial EEG connectivity, connectome gradient analysis and histopathology, we examined whether SEEG implantation is associated with large-scale network reorganisation (Larivière et al., 2024; Margulies et al., 2016) related to local electrode-induced tissue injury. The neurophysiological and imaging markers were chosen for their established clinical relevance in focal epilepsy: interictal intracranial-EEG functional connectivity relates to the epileptogenic zone and to postsurgical outcome (Bartolomei et al., 2017; Goodale et al., 2020; Lagarde et al., 2018), and structural connectome and gradient-based measures characterise temporal lobe epilepsy, relate to cognition, and predict seizure outcome after temporal lobe surgery (Bonilha et al., 2015; Fadaie et al., 2021; Gleichgerrcht et al., 2018; Larivière et al., 2020).

## 2 Case report

**Fig. 1:**
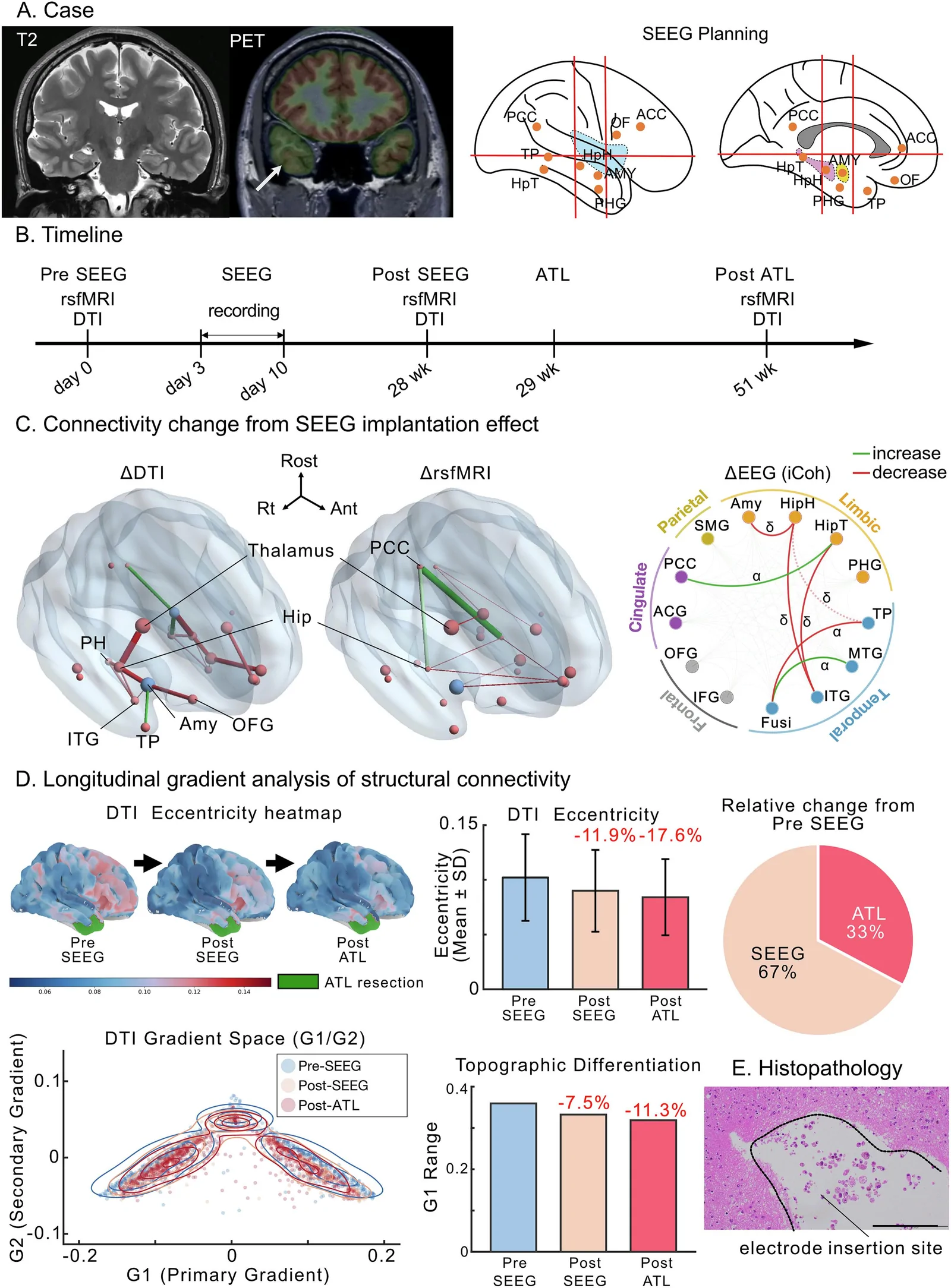
Multi-modal connectivity analysis after SEEG implantation in drug-resistant right temporal lobe epilepsy. (A) Clinical and SEEG implantation overview. Coronal T2-weighted MRI showed mild right hippocampal atrophy without clear hippocampal sclerosis, and FDG-PET showed slight hypometabolism in the right temporal pole (white arrow). Representative SEEG electrode trajectories sampled mesial temporal, lateral temporal, orbitofrontal and cingulate regions. AMY, amygdala; HpH, hippocampal head; HpT, hippocampal tail; PHG, parahippocampal gyrus; TP, temporal pole; OF, orbitofrontal cortex; ACC, anterior cingulate cortex; PCC, posterior cingulate cortex. (B) Timeline of data acquisition and treatment. MRI was acquired before SEEG, after SEEG implantation and after anterior temporal lobectomy. SEEG was recorded during intracranial monitoring. (C) Connectivity changes after SEEG implantation. Left, diffusion MRI structural connectivity changes; centre, resting-state fMRI functional connectivity changes; right, SEEG imaginary coherence changes between days 3 and 7. Red edges indicate decreased connectivity and green edges indicate increased connectivity; in the SEEG panel, Greek letters denote the frequency band of each edge (δ, delta; α, alpha). Across modalities, SEEG implantation was associated with decreased hippocampus–amygdala and fusiform gyrus–temporal pole connectivity, and increased hippocampus–posterior cingulate connectivity. In the SEEG panel, the hippocampus–amygdala decrease occurred in the delta band, whereas the fusiform gyrus–temporal pole decrease and the hippocampus–posterior cingulate increase occurred in the alpha band. Fusi, fusiform gyrus; ITG, inferior temporal gyrus; MTG, middle temporal gyrus; SMG, supramarginal gyrus; IFG, inferior frontal gyrus. (D) Longitudinal connectome gradient analysis. Cortical eccentricity heatmaps show progressive reduction in highly eccentric regions after SEEG and after lobectomy. Bar plots show reductions in mean diffusion MRI eccentricity and G1 range, indicating increased network integration and reduced topographic differentiation. The gradient-space plots show reduced dispersion of network nodes across timepoints. (E) Histopathology of an electrode trajectory site. Haematoxylin–eosin staining demonstrated lymphocytic infiltration and reactive gliosis adjacent to the electrode track, consistent with chronic inflammation. (For interpretation of the references to colour in this figure legend, the reader is referred to the web version of this article.)

A man in his 30s presented with a 4-year history of drug-resistant epilepsy despite treatment with levetiracetam, perampanel and lacosamide. His habitual seizures began with chest discomfort, déjà vu and fear, often progressing to impaired consciousness with oroalimentary automatisms. Scalp EEG showed right anterior temporal spikes and right temporal intermittent rhythmic delta activity, and ictal EEG suggested right temporal onset. 3 T MRI demonstrated mild right hippocampal atrophy without features of hippocampal sclerosis, while FDG-PET showed slight hypometabolism in the right temporal pole (Fig. 1A).

Because non-invasive evaluation suggested right temporal lobe epilepsy without a definitive structural lesion, SEEG was performed. Eight depth electrodes (Unique Medical Co., Ltd., Japan) were implanted to sample the right mesial temporal structures, lateral temporal cortex, orbitofrontal cortex, anterior cingulate cortex and posterior cingulate cortex. Interictal SEEG showed frequent epileptiform discharges in the mesial and lateral temporal regions. Ictal SEEG demonstrated seizure onset from the amygdala with propagation to the hippocampus, confirming right mesial temporal lobe epilepsy.

After electrode explantation, the patient reported marked seizure reduction: auras persisted, but seizures with impaired consciousness ceased. Six months later, right anterior temporal lobectomy with amygdalohippocampectomy was performed, resulting in ILAE outcome class 1a — completely seizure free since surgery, with no auras — at 6-month follow-up (Wieser et al., 2001).

## 3 Methods

3 T MRI, including T1-weighted imaging, diffusion MRI and resting-state fMRI, was acquired before SEEG implantation, 28 weeks after SEEG implantation (shortly before anterior temporal lobectomy) and 51 weeks after baseline, corresponding to 5.5 months after anterior temporal lobectomy (Fig. 1B). The antiseizure medication regimen (levetiracetam, perampanel and lacosamide) and doses were unchanged between the pre-SEEG and post-SEEG scans. SEEG was recorded continuously at 1000 Hz during intracranial monitoring.

Structural connectivity was estimated from diffusion MRI using MRtrix3 probabilistic tractography with SIFT2 weighting. Cortical and subcortical regions were parcellated using the Schaefer 400-parcel atlas and NextBrain atlas (Casamitjana et al., 2025; Schaefer et al., 2018). Resting-state fMRI was preprocessed using the CONN toolbox, including motion correction, bandpass filtering, confound regression and Fisher z-transformation of regional correlations. Because diffusion MRI and resting-state fMRI used different parcellations (Schaefer 400/NextBrain and Harvard–Oxford, respectively), cross-modal comparisons were restricted to anatomically corresponding region pairs.

SEEG connectivity was quantified using imaginary coherence, which is robust to volume-conduction artefacts (Nolte et al., 2004), a metric that has also been applied to resting-state SEEG to help localise epileptogenic regions (Goodale et al., 2020). 15–30 min of artefact-free interictal putative N3 (slow-wave) sleep (video-confirmed; Appendix S1, Section S4) were analysed for each timepoint. Connectivity was calculated across delta, theta, alpha, beta and gamma bands. Because antiseizure medications were withdrawn during days 1–2 and then restarted, intra-recording changes were assessed between days 3 and 7 to reduce pharmacological confounding. Doses were unchanged between days 3 and 7, a condition under which SEEG imaginary coherence has been shown to remain stable across consecutive monitoring days (Paulo et al., 2022). All five canonical bands were analysed; the edge-level changes reported below occurred in the delta and alpha bands.

Connectome gradients were computed from connectivity matrices using diffusion map embedding with a normalised angle kernel and 90% sparsity threshold (Larivière et al., 2024; Margulies et al., 2016). Eccentricity was defined as each region's Euclidean distance from the centroid in four-dimensional (G1–G4) gradient space, with lower values indicating greater integration; the G1 range (spread along the principal gradient, G1) indexed topographic differentiation. Given the single-case design, ROI- and edge-wise paired statistics and cross-modal concordance, rather than subject-level inferential tests, were used to evaluate whether observed changes were spatially systematic and consistent across regions (Appendix S1, Section S6).

Resected tissue around electrode trajectories was examined with haematoxylin–eosin staining by a board-certified neuropathologist.

## 4 Results

Longitudinal multi-modal connectivity analysis revealed convergent network changes after SEEG implantation (Fig. 1C). Diffusion MRI showed prominent structural connectivity decreases centred on the right mesial temporal lobe, including the hippocampus, parahippocampal gyrus and entorhinal cortex. Decreased connectivity also extended along the fusiform gyrus–temporal pole pathway, corresponding anatomically to electrode trajectories. In contrast, hippocampus–posterior cingulate connectivity increased. Resting-state fMRI demonstrated a concordant pattern, with reduced hippocampus–temporal pole connectivity and increased posterior cingulate–hippocampus connectivity, suggesting enhanced default mode network engagement. Contralateral increases in left amygdala and inter-hemispheric hippocampal connectivity were also observed.

Intracranial EEG connectivity showed parallel temporal dynamics during monitoring. Between days 3 and 7, imaginary coherence decreased most prominently within the limbic network and in the delta band, consistent with reduced pathological synchronisation. Edge-level analysis showed decreased hippocampus–amygdala connectivity in the delta band and decreased fusiform–temporal pole connectivity in the alpha band, whereas hippocampus–posterior cingulate connectivity increased in the alpha band. Thus, a broadly concordant pattern of limbic disconnection with increased hippocampus–posterior cingulate connectivity was seen across all three modalities.

Connectome gradient analysis further supported SEEG-associated network reorganisation (Fig. 1D). Mean diffusion MRI eccentricity decreased by 11.9% (95% CI 9.9–13.7%; Cohen's d = 0.44) after SEEG and by 17.6% (95% CI 15.6–19.7%; d = 0.58) after anterior temporal lobectomy relative to baseline. These effect sizes were small-to-moderate, and the reduction was graded across the two interventions, consistent with a progressive shift toward greater network integration. Eccentricity heatmaps showed that highly eccentric cortical regions became less prominent over time, particularly after SEEG implantation. Overall, 67% (95% bootstrap CI 58–77%) of the total pre-SEEG-to-post-lobectomy eccentricity change had already occurred after SEEG implantation.

In the gradient space, network nodes became less dispersed across timepoints, consistent with reduced topographic differentiation and greater integration. The post-SEEG and post-lobectomy principal gradient (G1) maps were highly spatially correlated (Pearson *r* = 0.90). Because gradient maps themselves are more reproducible than scalar gradient-dispersion summaries such as eccentricity, this spatial concordance — rather than the eccentricity magnitude alone — indicates that the network topology established after SEEG resembled that observed after definitive surgery. The G1 range, reflecting differentiation along the principal connectivity gradient, decreased by 7.5% after SEEG and by 11.3% after lobectomy.

Histopathological examination of resected tissue around electrode trajectories demonstrated lymphocytic infiltration and reactive gliosis, consistent with chronic inflammation at electrode insertion sites (Fig. 1E).

## 5 Discussion

In this single case, SEEG implantation was associated with measurable, multi-modal network changes that paralleled clinical seizure reduction. The key finding was the convergence of these connectivity changes across diffusion MRI, resting-state fMRI and intracranial EEG, making a modality-specific artefact unlikely. Rather, these findings are compatible with electrode-associated modification of the temporal-limbic network, although a single case cannot establish this directly.

The observed pattern is biologically plausible. Decreased hippocampus–amygdala connectivity is consistent with disruption of the mesial temporal seizure network, while reduced fusiform–temporal pole connectivity may reflect interruption of temporal propagation pathways along electrode trajectories. Conversely, increased hippocampus–posterior cingulate connectivity suggests greater engagement of the default mode network, which is associated with preserved memory function in mesial temporal lobe epilepsy (McCormick et al., 2013). The SEEG finding of reduced delta-band limbic synchronisation supports attenuation of pathological long-range coupling, increasingly considered relevant to network-guided epilepsy treatment (Courtiol et al., 2020; Piper et al., 2022). This interpretation is consistent with the observation that epileptogenic regions show elevated interictal functional connectivity on resting-state SEEG (Goodale et al., 2020), so that the reduction observed here after implantation is compatible with attenuation of a pathologically elevated state. Delta-band change is of particular relevance in this patient, whose scalp EEG showed temporal intermittent rhythmic delta activity, a recognised marker of mesial temporal lobe epilepsy (Di Gennaro et al., 2003); moreover, among patients undergoing SEEG-guided thermocoagulation, responders showed reductions in interictal delta- and alpha-band functional connectivity that non-responders did not (Simula et al., 2023).

Gradient analysis provided a complementary topological perspective. Mean eccentricity decreased progressively across the two interventions, indicating reduced segregation from the global network centroid. We interpret this cautiously: the effect sizes were small-to-moderate, and the diffusion MRI connectomes underlying these gradients show non-trivial test–retest variability (≈3–24%) in prior reports (Buchanan et al., 2014). Three features nonetheless make a purely incidental measurement fluctuation a less parsimonious explanation. First, the change scaled with the extent of intervention, with 67% of the total change already present after SEEG, arguing against random scan-to-scan variability. Second, at the more reproducible map level, the post-SEEG and post-lobectomy G1 maps were highly correlated, indicating reorganisation in the same direction as definitive surgery. Third, in a normative comparison processed through the identical pipeline, the patient's longitudinal eccentricity reduction exceeded the between-subject variability of a healthy reference cohort and shifted monotonically toward the healthy distribution (Appendix S1, Section S6). The accompanying reduction in G1 range is consistent with partial normalisation of the expanded gradients described in temporal lobe epilepsy (Larivière et al., 2024). Together, these observations increase the plausibility of an electrode-associated network effect, although coincidental co-occurrence cannot be excluded in a single case.

Our longitudinal findings also align with cohort evidence that the connectome is outcome-relevant in temporal lobe surgery. Presurgical structural network architecture predicts postoperative seizure freedom after temporal lobectomy (Bonilha et al., 2015), including with deep-learning approaches (Gleichgerrcht et al., 2018), and manifold-scale functional connectome contractions likewise predict surgical outcome (Larivière et al., 2020). Temporal lobe resection itself reorganises the remaining structural connectome, and the extent of this reorganisation relates to seizure outcome (Taylor et al., 2018). The present case adds a within-patient, longitudinal perspective to this largely group-level literature, following the same connectome metrics across both the implantation and the resection. A longitudinal functional connectome study before and after anterior temporal lobectomy similarly found that network reorganisation differed between seizure-free and non-seizure-free patients (Liao et al., 2016), although that comparison was made between groups rather than within a single patient.

Histopathology demonstrated lymphocytic infiltration and reactive gliosis around electrode trajectories, indicating local tissue injury and supporting the interpretation that the implantation effect may represent a network-level consequence of electrode-induced micro-lesions rather than a purely clinical fluctuation. This mechanism is conceptually consistent with SEEG-guided radiofrequency thermocoagulation, in which targeted disruption of epileptic networks can reduce seizures (Bourdillon et al., 2017; Campbell et al., 2026).

Whether the observed network changes were a cause, a consequence, or an epiphenomenon of the seizure reduction cannot be settled in a single case. It is helpful to distinguish three causal links: (i) implantation to seizure reduction; (ii) implantation to network change; and (iii) the relationship between the network change and the seizure reduction. We evaluated these against the nine causality criteria that Asano et al. (2013) adapted from Hill for the presurgical evaluation of epilepsy.

The temporal relationship is satisfied for links (i) and (ii): both the clinical improvement and the connectivity changes were absent at baseline and were observed only after electrode insertion, and the intracranial-EEG changes were already present during monitoring, between days 3 and 7. A biological gradient is supported, in that the magnitude of change scaled with the extent of intervention: 67% of the total eccentricity change was already present after implantation alone, with further change after lobectomy. Replication of findings is satisfied for link (i), since seizure reduction after intracranial implantation alone has been reported independently across centres (Bottan et al., 2023; Katariwala et al., 2001; Kaur et al., 2019; Percy et al., 2020), but not for link (ii), whose network correlates are described here in a single patient at a single centre. Coherence is supported, as the direction of change is consistent with the accepted view that disruption of mesial temporal and limbic pathways can reduce seizures, and with the expanded connectome gradients described in temporal lobe epilepsy (Larivière et al., 2024). Biological plausibility is supported by the histopathological finding of lymphocytic infiltration and reactive gliosis along the electrode trajectories, which provides a tangible lesional substrate. Analogy is supported by established disconnective procedures whose therapeutic rationale is precisely the alteration of network connectivity: multiple subpial transection was designed on the premise that epileptogenic discharge requires horizontal intracortical interaction, and severs tangential fibres while preserving vertical connections (Morrell et al., 1989; Spencer et al., 2002), and SEEG-guided radiofrequency thermocoagulation reduces seizures by disrupting epileptic networks (Bourdillon et al., 2017; Campbell et al., 2026). By contrast, the strength of association cannot be quantified in a single patient, as there is no control group and no confidence interval for the clinical association; the normative comparison with a healthy reference cohort provides a scale reference rather than a measure of association. Specificity is not met, since the observed changes are not specific to seizure reduction and cannot be weighed against alternative outcomes in one patient, and experimental evidence is absent, as no controlled or randomised manipulation was performed. Five criteria are therefore supported, one is satisfied for link (i) only, and three are not met or not assessable.

Link (iii) is the least secure of the three. Our data show that network changes followed implantation, but not that they mediated the seizure reduction; reverse causation remains possible, in that a reduced seizure and interictal discharge burden may itself remodel networks. Nevertheless, the simplest explanation is that the network change contributed to the seizure reduction. The implanted depth electrodes physically traversed the mesial and lateral temporal structures that generated this patient's seizures, and histopathology confirmed tissue injury along those trajectories, so that a functional consequence for the temporal-limbic network is to be expected rather than incidental. The premise that altering network connectivity reduces seizures is, moreover, the design principle of several established procedures (Bourdillon et al., 2017; Morrell et al., 1989), and longitudinal studies indicate that connectivity change after an intervention tracks seizure outcome: the degree of network reorganisation during responsive neurostimulation scales with seizure reduction (Khambhati et al., 2021), postoperative rather than preoperative connectivity distinguishes seizure-free from non-seizure-free patients after temporal lobe surgery (Akbarian et al., 2024), and responders to SEEG-guided thermocoagulation show interictal connectivity reductions that non-responders do not (Simula et al., 2023). What a single case cannot do is establish the direction and the magnitude of that contribution here; we therefore present this report as hypothesis-generating.

This report has limitations. As a single case, it cannot establish generalisability. Post-SEEG imaging was obtained after electrode removal and may therefore reflect a chronic tissue response. Medication changes, perioperative factors and spontaneous seizure fluctuation cannot be fully excluded. With a single acquisition per timepoint, scan–rescan reliability could not be established, so the eccentricity findings represent convergent support rather than independent confirmation. Nevertheless, the convergence across longitudinal diffusion MRI, resting-state fMRI, intracranial EEG and histopathology — limbic disconnection with enhanced hippocampus–posterior cingulate connectivity and partial topological normalisation — provides a plausible network-level explanation for the implantation effect.

## Ethics and consent

This study was approved by the Ethics Committee of Keio University School of Medicine (Approval No. 20241119; approved 19 November 2024) and was conducted in accordance with the Declaration of Helsinki. The patient provided written informed consent to participate and for publication of this case report and the accompanying images. Written consent has been retained by the authors.

## Funding

This research did not receive any specific grant from funding agencies in the public, commercial, or not-for-profit sectors.

## Declaration of generative AI and AI-assisted technologies in the manuscript preparation process

During the preparation of this work the authors used Anthropic Claude in order to polish the English language. After using this tool, the authors reviewed and edited the content as needed and take full responsibility for the content of the published article.

## Declaration of competing interest

The authors declare that they have no known competing financial interests or personal relationships that could have appeared to influence the work reported in this paper.

## Appendix A Supplementary data

Supplementary material: Appendix S1. Supplementary Methods (MRI acquisition; diffusion MRI; resting-state fMRI; intracranial EEG connectivity; connectome gradient analysis; statistical considerations; histopathology).

## References

[bb0005] Akbarian B., Sainburg L.E., Janson A. (2024). Association between postsurgical functional connectivity and seizure outcome in patients with temporal lobe epilepsy. Neurology.

[bb0010] Asano E., Brown E.C., Juhász C. (2013). How to establish causality in epilepsy surgery. Brain Dev..

[bb0015] Bartolomei F., Lagarde S., Wendling F. (2017). Defining epileptogenic networks: contribution of SEEG and signal analysis. Epilepsia.

[bb0020] Bonilha L., Jensen J.H., Baker N. (2015). The brain connectome as a personalized biomarker of seizure outcomes after temporal lobectomy. Neurology.

[bb0025] Bottan J.S., Alshahrani A., Gilmore G. (2023). Lack of spontaneous typical seizures during intracranial monitoring with stereo-electroencephalography. Epileptic Disord..

[bb0030] Bourdillon P., Isnard J., Catenoix H. (2017). Stereo electroencephalography–guided radiofrequency thermocoagulation (SEEG-guided RF-TC) in drug-resistant focal epilepsy: results from a 10-year experience. Epilepsia.

[bb0035] Buchanan C.R., Pernet C.R., Gorgolewski K.J. (2014). Test–retest reliability of structural brain networks from diffusion MRI. NeuroImage.

[bb0040] Campbell B., Neal A., Cockle E. (2026). Stereo-electroencephalography-guided cross-electrode radiofrequency thermocoagulation in focal epilepsy: a review of current methodologies and outcomes. Epilepsia.

[bb0045] Casamitjana A., Mancini M., Robinson E. (2025). A probabilistic histological atlas of the human brain for MRI segmentation. Nature.

[bb0050] Courtiol J., Guye M., Bartolomei F. (2020). Dynamical mechanisms of interictal resting-state functional connectivity in epilepsy. J. Neurosci..

[bb0055] Di Gennaro G., Quarato P.P., Onorati P. (2003). Localizing significance of temporal intermittent rhythmic delta activity (TIRDA) in drug-resistant focal epilepsy. Clin. Neurophysiol..

[bb0060] Fadaie F., Lee H.M., Caldairou B. (2021). Atypical functional connectome hierarchy impacts cognition in temporal lobe epilepsy. Epilepsia.

[bb0065] Gleichgerrcht E., Munsell B., Bhatia S. (2018). Deep learning applied to whole-brain connectome to determine seizure control after epilepsy surgery. Epilepsia.

[bb0070] Goodale S.E., González H.F.J., Johnson G.W. (2020). Resting-state SEEG may help localize epileptogenic brain regions. Neurosurgery.

[bb0075] Jayakar P., Gotman J., Harvey A.S. (2016). Diagnostic utility of invasive EEG for epilepsy surgery: indications, modalities, and techniques. Epilepsia.

[bb0080] Katariwala N.M., Bakay R.A.E., Pennell P.B. (2001). Remission of intractable partial epilepsy following implantation of intracranial electrodes. Neurology.

[bb0085] Kaur M., Szaflarski J.P., Ver Hoef L. (2019). Long-term seizure freedom following intracranial sEEG monitoring: therapeutic benefit of a diagnostic technique. Epilepsy Behav. Rep..

[bb0090] Khambhati A.N., Shafi A., Rao V.R. (2021). Long-term brain network reorganization predicts responsive neurostimulation outcomes for focal epilepsy. Sci. Transl. Med..

[bb0095] Lagarde S., Roehri N., Lambert I. (2018). Interictal stereotactic-EEG functional connectivity in refractory focal epilepsies. Brain.

[bb0100] Larivière S., Weng Y., Vos de Wael R. (2020). Functional connectome contractions in temporal lobe epilepsy: microstructural underpinnings and predictors of surgical outcome. Epilepsia.

[bb0105] Larivière S., Park B.Y., Royer J. (2024). Connectome reorganization associated with temporal lobe pathology and its surgical resection. Brain.

[bb0110] Liao W., Ji G.J., Xu Q. (2016). Functional connectome before and following temporal lobectomy in mesial temporal lobe epilepsy. Sci. Rep..

[bb0115] Margulies D.S., Ghosh S.S., Goulas A. (2016). Situating the default-mode network along a principal gradient of macroscale cortical organization. Proc. Natl. Acad. Sci. U. S. A..

[bb0120] McCormick C., Quraan M., Cohn M. (2013). Default mode network connectivity indicates episodic memory capacity in mesial temporal lobe epilepsy. Epilepsia.

[bb0125] Morrell F., Whisler W.W., Bleck T.P. (1989). Multiple subpial transection: a new approach to the surgical treatment of focal epilepsy. J. Neurosurg..

[bb0130] Nolte G., Bai O., Wheaton L., Mari Z., Vorbach S., Hallett M. (2004). Identifying true brain interaction from EEG data using the imaginary part of coherency. Clin. Neurophysiol..

[bb0135] Paulo D.L., Wills K.E., Johnson G.W. (2022). SEEG functional connectivity measures to identify epileptogenic zones: stability, medication influence, and recording condition. Neurology.

[bb0140] Percy J., Zaveri H.P., Duckrow R.B. (2020). Beyond implantation effect? Long-term seizure reduction and freedom following intracranial monitoring without additional surgical interventions. Epilepsy Behav..

[bb0145] Piper R.J., Richardson R.M., Worrell G. (2022). Towards network-guided neuromodulation for epilepsy. Brain.

[bb0150] Schaefer A., Kong R., Gordon E.M. (2018). Local-global parcellation of the human cerebral cortex from intrinsic functional connectivity MRI. Cereb. Cortex.

[bb0155] Simula S., Garnier E., Contento M. (2023). Changes in local and network brain activity after stereotactic thermocoagulation in patients with drug-resistant epilepsy. Epilepsia.

[bb0160] Spencer S.S., Schramm J., Wyler A. (2002). Multiple subpial transection for intractable partial epilepsy: an international meta-analysis. Epilepsia.

[bb0165] Suresh S., Sweet J., Fastenau P.S. (2015). Temporal lobe epilepsy in patients with nonlesional MRI and normal memory: an SEEG study. J. Neurosurg..

[bb0170] Taylor P.N., Sinha N., Wang Y. (2018). The impact of epilepsy surgery on the structural connectome and its relation to outcome. NeuroImage Clin..

[bb0175] Wieser H.G., Blume W.T., Fish D. (2001). ILAE commission report. Proposal for a new classification of outcome with respect to epileptic seizures following epilepsy surgery. Epilepsia.

