# Supplementary material for: Multi-modal connectome analysis of the stereoelectroencephalography implantation effect: a case report
Source: Clin Neurophysiol Pract. 2026 Sep 12;11:782–7. doi: 10.1016/j.cnp.2026.09.002 (PMC13594891; doi:10.1016/j.cnp.2026.09.002)
Supplement: Supplementary file 1 [file mmc1.docx]

# Appendix S1. Supplementary Methods

## S1. Magnetic resonance imaging (MRI) acquisition and preprocessing

3T MRI was acquired at three timepoints: pre-SEEG (day 0), post-SEEG (28 weeks; approximately 6 months after electrode explantation), and post-anterior temporal lobectomy (ATL; 51 weeks; approximately 5.5 months after surgery). The acquisition protocol at each timepoint included T1-weighted structural imaging, multi-shell diffusion MRI (32 directions) and resting-state functional MRI (rs-fMRI). The antiseizure medication regimen (levetiracetam 3000 mg/day, perampanel 6 mg/day, lacosamide 300 mg/day) was unchanged between the pre-SEEG and post-SEEG scans, minimising pharmacological confounding of MRI-based connectivity changes.

## S2. Structural connectivity (diffusion MRI)

Diffusion MRI preprocessing was performed in MRtrix3. The pipeline included Marchenko-Pastur PCA denoising, Gibbs ringing removal, motion and eddy-current correction (FSL eddy), bias field correction and brain masking. Multi-shell multi-tissue constrained spherical deconvolution (MSMT-CSD) was used to estimate fiber orientation distributions for white matter, grey matter and cerebrospinal fluid. Anatomically constrained whole-brain tractography was performed using the iFOD2 algorithm with 10 million streamlines. Spherical-deconvolution informed filtering of tractograms (SIFT2) was applied to obtain quantitative edge weights proportional to apparent fiber density.

Cortical regions were parcellated using the Schaefer 400-region functional atlas (7-network solution). Subcortical and medial temporal regions were parcellated using the NextBrain probabilistic histological atlas, yielding approximately 840 regions of interest (ROIs) in total. To match ROIs across timepoints in the post-ATL session, voxels overlapping the resection cavity were excluded from both the tractography seeding mask and the parcellation, leaving 818 ROIs. This same 818-ROI set was used at all timepoints for paired analyses.

## S3. Functional connectivity (resting-state fMRI)

rs-fMRI preprocessing was performed using the CONN functional connectivity toolbox. Steps included realignment, slice-timing correction, ART-based outlier detection, normalisation to MNI152 space, smoothing (8 mm FWHM) and denoising using the anatomical CompCor framework (5 principal components each from white matter and CSF, plus 6 motion parameters and their first derivatives). A bandpass filter (0.008–0.09 Hz) was applied. Region-to-region functional connectivity was quantified as Fisher-z transformed Pearson correlation between mean ROI BOLD time series. The Harvard–Oxford atlas was used for cortical and subcortical parcellation, supplemented with default-mode network ROIs (medial prefrontal cortex, posterior cingulate cortex) from the CONN atlas.

## S4. Intracranial EEG (SEEG) connectivity

Continuous SEEG was recorded at 1,000 Hz during the 7-day intracranial monitoring period using a Nihon Kohden EEG acquisition system. For each recording day, artefact-free interictal data from the patient’s habitual sleep window were screened for N3 (slow-wave sleep) epochs as described below, and approximately 15–30 minutes of N3 sleep per recording day were retained for connectivity analysis.

Sleep stage was assigned epoch-by-epoch using a custom automated classifier implemented in MATLAB that operates directly on the intracranial signal (scalp EEG with electrooculography and electromyography was not available during SEEG monitoring). For each 30-second epoch, multitaper power spectral density was computed for selected intracranial channels (anterior cingulate gyrus, orbitofrontal gyrus and bilateral hippocampal head/tail), and band powers (slow-delta 0.5–2 Hz, delta 1–4 Hz, theta 4–8 Hz, alpha 8–13 Hz, beta 13–30 Hz, sigma 11–16 Hz) were z-scored across the recording. Sleep spindles (11–16 Hz Hilbert envelope above mean + 2 SD) were also quantified in cingulate and orbitofrontal channels. Five sleep stages (Wake, N1, N2, N3 and REM) were then classified by selecting the maximum of five composite scores derived from these features; the N3 score combined high cingulate and orbitofrontal delta power with suppressed alpha and beta power. The resulting epoch-by-epoch stage assignments were temporally smoothed with a 3-epoch median filter. The N3 stage was chosen because of (i) the relative stability of cortical activity during slow-wave sleep, (ii) the lower contribution of behavioural state-related variability, and (iii) precedent in epilepsy iEEG connectivity studies that sample N3 to reduce vigilance-state confounding.

Because the standard American Academy of Sleep Medicine (AASM) staging is defined on simultaneous scalp EEG with electrooculography and electromyography, which were not available during SEEG monitoring, the automated stage labels obtained above were **verified by clinician review of the simultaneously recorded synchronised video** by the responsible physician (T.S.). Specifically, the time intervals labelled as N3 by the classifier were checked against the video for behavioural correlates of deep sleep (absence of body movement, eyes closed throughout, regular respiratory rhythm and absence of rapid eye movements that would suggest REM). Epochs whose classifier label was not supported by the corresponding video were excluded from the analysis. Only epochs whose automated N3 label was confirmed by video review were retained for connectivity computation. We note that this combined automated-plus-video procedure does not strictly conform to AASM criteria, which require scalp EEG with EOG and EMG; rather, it is a pragmatic adaptation for the SEEG-only setting that combines an iEEG-based automated classifier with bedside-video confirmation by the treating clinician. We therefore use the label “N3” throughout to refer to **slow-wave-sleep-like epochs identified by this combined automated and video-confirmed iEEG procedure**, and not to assert formal AASM-compliant N3 sleep.

Bipolar montages were derived from neighbouring electrode contacts to minimise volume-conduction artefacts. Signals were high-pass filtered at 0.5 Hz, low-pass filtered at 100 Hz, and notch filtered at 50 Hz. Contacts located in white matter or showing persistent artefacts were excluded.

Connectivity was quantified using **imaginary coherence (iCoh)** computed as:

iCoh_xy(f) = | Im( ⟨S_xy(f)⟩ ) | / sqrt( ⟨|H_x(f)|²⟩ · ⟨|H_y(f)|²⟩ )

where *S_xy(f)* is the cross-spectral density between channels *x* and *y* at frequency *f*, *H_x(f)* and *H_y(f)* are the corresponding short-time analytic signals, and ⟨·⟩ denotes averaging over short overlapping segments within the epoch (Nolte et al., *Clin Neurophysiol* 2004). Because iCoh discards the zero-phase component of the cross-spectrum, it is robust to spurious coupling caused by volume conduction. The spectral computations used multitaper estimation (MATLAB pmtm, 4 tapers) and mscohere (Hamming windowed segments, 50% overlap), and iCoh was averaged over each of five canonical frequency bands (delta 1–4 Hz, theta 4–8 Hz, alpha 8–13 Hz, beta 13–30 Hz, gamma 30–80 Hz). The full implementation was an in-house MATLAB pipeline running on MATLAB R2024a with the Signal Processing Toolbox and the Parallel Computing Toolbox.

Antiseizure medications were tapered during days 1–2 and re-established from day 3 to provoke seizures during clinical monitoring. To remove this pharmacological confound from the connectivity-change analyses, intra-recording dynamic changes were assessed between **day 3 and day 7**, when steady-state pharmacological conditions were maintained.

## S5. Connectome gradient analysis

For each timepoint, a region-by-region structural connectome (DTI, 818 × 818 ROIs) was used to derive connectivity gradients with the BrainSpace toolbox, following the approach of Larivière et al. (*Brain* 2024). The connectome was thresholded at 90% sparsity (keeping the strongest 10% of edges), and a row-wise normalised angle kernel was applied to produce an affinity matrix. Diffusion-map embedding (α = 0.5, diffusion time t = 0) decomposed the affinity matrix into orthogonal connectivity gradients G1, G2, …, G_n, ordered by explained variance. G1 corresponds to the principal axis of macroscale connectivity organisation. G2 was used to evaluate complementary directions of gradient differentiation (e.g. sensorimotor–visual separation). Post-SEEG and post-ATL gradient maps were aligned to the pre-SEEG reference using Procrustes alignment to permit ROI-wise paired comparison across timepoints.

For each ROI, **eccentricity** was defined as the Euclidean distance from the centroid of the gradient cloud in the four-dimensional space spanned by G1–G4:

Eccentricity_i = sqrt( (G1_i − G1̄)² + (G2_i − G2̄)² + (G3_i − G3̄)² + (G4_i − G4̄)² )

Lower eccentricity indicates that a region sits closer to the global gradient centroid, i.e. greater functional integration. The **G1 range** (max(G1) − min(G1)) was used as a single-number index of topographic differentiation along the principal connectivity axis. The fraction of total Pre→Post-ATL change captured by the SEEG step (the “SEEG fraction”) was computed as:

SEEG-fraction = (Mean Pre→Post-SEEG Δ-eccentricity) / (Mean Pre→Post-ATL Δ-eccentricity) × 100%

## S6. Statistical considerations

This study is a **single-case longitudinal analysis**. Because each measurement timepoint provides one observation per subject, traditional group-level inferential statistics are not applicable, and a within-subject test–retest MRI of this patient acquired in the absence of any intervention is not available. We therefore cannot estimate the within-subject measurement variability (test–retest reliability) of the connectome gradient metric directly from this patient’s own data. Published test–retest data for DTI-based structural brain networks indicate that within-subject reliability of network metrics is moderate to high but is metric-dependent (Buchanan et al., *NeuroImage* 2014); we cite this as context but do not use those values as a quantitative noise floor in this single case.

To support interpretation of the observed longitudinal changes in the absence of within-subject test–retest data, the within-modality statistical analyses reported below are framed as descriptive assessments of **whether the observed changes are spatially systematic and consistent across regions**, and are complemented by an empirical normative comparison with an independent healthy reference cohort that provides a between-subject variability scale for the same metric (subsection (iii) below). Three complementary strategies were used:

**(i) Spatial systematicity at the ROI / edge level.** For the DTI gradient analysis, paired Wilcoxon signed-rank tests were performed across the 818 ROIs comparing eccentricity between timepoints (Pre-SEEG vs Post-SEEG; Pre-SEEG vs Post-ATL). A small p-value here indicates that, across regions, the direction of eccentricity change is systematically biased rather than randomly scattered; it does **not** test whether the magnitude exceeds within-subject test–retest variability. Cohen’s *d* (paired) was calculated as a standardised effect size of the regional shift. 95% confidence intervals (CIs) for the mean eccentricity change across ROIs were obtained by ROI-level non-parametric bootstrap with 10,000 resamples. For the iCoh-based SEEG analyses, descriptive metrics (mean, standard deviation, %-increase across edges, |Δ|/SD effect-size ratio) were derived from the per-band edge distributions, and a binomial test (against H₀ = 50%) was used to assess whether the proportion of edges that increased differed from a chance distribution. For DTI and rs-fMRI edge-level connectivity, the absolute and relative changes (Post − Pre, (Post − Pre) / Pre × 100%) are reported.

**(ii) Cross-modal and procedural concordance.** Independent observation of the same directional change across DTI, rs-fMRI and SEEG (cross-modal concordance), and parallel directionality between the Post-SEEG and Post-ATL 818-ROI gradient maps (procedural concordance), provide a complementary, model-free indication that the observed changes are unlikely to reflect modality-specific noise. Spatial Pearson correlation between the Post-SEEG and Post-ATL G1 gradient maps was used to quantify procedural concordance.

**(iii) Comparison with a healthy reference cohort.** To provide a quantitative normative scale for the magnitude of the observed longitudinal change — although a within-subject test–retest measurement was not available — we additionally processed a healthy adult cohort (n ≈ 200, ages 22–35) from the Human Connectome Project (HCP; Van Essen et al., *NeuroImage* 2013) accessed via the ENIGMA Toolbox (Larivière et al., *Nat Methods* 2021), using the identical BrainSpace pipeline (normalised-angle kernel, 90% sparsity, diffusion-map embedding) as for the patient analysis. For this HCP cohort, DTI structural connectomes parcellated with the Schaefer 400 cortical atlas (Schaefer et al., *Cereb Cortex* 2018) yielded a mean cortical eccentricity of 0.060 ± 0.015 (mean ± between-subject SD across subjects). The corresponding eccentricity values in the present patient were 0.102 at Pre-SEEG (z = +2.87 relative to the HCP cohort), 0.090 at Post-SEEG (z = +2.04) and 0.084 at Post-ATL (z = +1.64). The Pre-SEEG-to-Post-ATL change in this patient (Δ ≈ −0.018) is larger than the between-subject SD observed in the HCP cohort (0.015), and the z-score trajectory shifts monotonically toward the healthy reference distribution (+2.87 → +2.04 → +1.64). Although this comparison does not substitute for direct within-subject test–retest data, the broad picture — that the longitudinal change exceeds the natural between-subject variability of the same metric in a large healthy cohort and moves the patient toward the healthy distribution — supports the interpretation that the observed change is unlikely to be fully explained by random measurement variability.

We acknowledge the following caveats of this normative comparison: (1) the HCP cohort provides between-subject variability, not within-subject test–retest variability, although the two are typically related (between-subject SD is generally not smaller than within-subject test–retest SD); (2) MRI acquisition platforms, scanners and protocols differ between our institution and HCP; and (3) the HCP analysis used a cortical-only Schaefer 400 atlas whereas the patient analysis incorporated both Schaefer 400 cortical and NextBrain subcortical ROIs (818 in total), and eccentricity values are atlas-dependent. The absolute z-scores should therefore be interpreted as a coarse scale reference rather than as quantitative inferential statistics, while the directional trajectory toward the healthy reference distribution is robust to the atlas mismatch.

No correction for multiple comparisons was applied to the within-modality paired tests, as these are used as descriptive complements to the directional findings rather than to support multiple parallel hypotheses. p-values are reported as point estimates and should be interpreted in the context of the single-case design and the limitations described above.

## S7. Histopathological examination

Anterior temporal lobectomy specimens were fixed in 10% neutral buffered formalin, embedded in paraffin, sectioned at 4 µm and stained with haematoxylin and eosin (H&E). Sections were screened by a board-certified neuropathologist (Y.N.) for histopathological abnormalities, including signs of hippocampal sclerosis (per ILAE classification), focal cortical dysplasia and inflammation. Special attention was paid to identifying electrode insertion trajectories, which were recognisable as linear regions of tissue disruption with adjacent reactive gliosis and lymphocytic infiltration.

## Supplementary References

S1. Nolte G, Bai O, Wheaton L, Mari Z, Vorbach S, Hallett M. Identifying true brain interaction from EEG data using the imaginary part of coherency. *Clin Neurophysiol* 2004;115:2292–307. doi:10.1016/j.clinph.2004.04.029

S2. Buchanan CR, Pernet CR, Gorgolewski KJ, Storkey AJ, Bastin ME. Test–retest reliability of structural brain networks from diffusion MRI. *NeuroImage* 2014;86:231–43. doi:10.1016/j.neuroimage.2013.09.054

S3. Van Essen DC, Smith SM, Barch DM, Behrens TEJ, Yacoub E, Ugurbil K; WU-Minn HCP Consortium. The WU-Minn Human Connectome Project: an overview. *NeuroImage* 2013;80:62–79. doi:10.1016/j.neuroimage.2013.05.041

S4. Larivière S, Paquola C, Park BY, et al. The ENIGMA Toolbox: multiscale neural contextualization of multisite neuroimaging datasets. *Nature Methods* 2021;18:698–700. doi:10.1038/s41592-021-01186-4

S5. Schaefer A, Kong R, Gordon EM, et al. Local-global parcellation of the human cerebral cortex from intrinsic functional connectivity MRI. *Cerebral Cortex* 2018;28:3095–3114. doi:10.1093/cercor/bhx179
